# The association between Dental Fluorosis and COL1A2 gene polymorphism among a Tunisian Population

**DOI:** 10.1186/s12903-024-04086-z

**Published:** 2024-03-22

**Authors:** Rim Kallala, Afef Slimani, Yosra Gassara, Behaeddin Garrach, Sawssen Chouchen, Hajer Foddha, Asma Rouis, Aberraouf kenani

**Affiliations:** 1 Faculty of dental Medicine Monastir, University of Monastir, Monastir, 5000 Tunisia; 2 Laboratory of Occlusodontics and Ceramic Prostheses, Monastir, LR16ES15, 5000 Tunisia; 3 Faculty of Medicine Monastir Tunisia, University of Monastir, Monastir, 5000 Tunisia; 4 laboratory of Environment, Inflammation, Signaling and Pathologies, Monastir, LR 18ES40, 5000 Tunisia; 5 Faculty of Pharmacy, University of Monastir, Monastir, 5000 Tunisia; 6 Hematology department, Fattouma Bourguiba University Hospital, Monastir, 5000 Tunisia; 7 Laboratory of human genome and multifactorial diseases (LR12ES07), Faculty of Pharmacy, University of Monastir, University of Monastir, Monastir, 5000 Tunisia; 8 Stomatology department, Hospital of Jammel, Monastir, 5000 Tunisia

**Keywords:** Esthetic, Fluorides, Fluorine, Tooth, North Africa, Single nucleotide polymorphism (SNP)

## Abstract

Dental fluorosis (DF) is a prevalent developmental defect of tooth enamel caused by exposure to excessive fluoride, with the severity dependent on various factors. This study aimed to investigate the association between DF and a specific genetic polymorphism (rs412777) in the COL1A2 gene among a Tunisian population. A case-control study was conducted from July to November 2022, involving a total of 95 participants including 51 cases and 44 controls. Dental examinations and genetic analysis were performed to assess the relationship between the COL1A2 gene polymorphism and DF.

The results of allelic distribution revealed that A allele carriers were significantly protected against (DF) when compared to those with the C allele (C vs. A, *p* = 0.001; OR = 0.375 (0.207–0.672)). This suggests a strong correlation between the presence of the C allele and the risk of developing DF. Additionally, significant association between the CC genotype of rs412777 and an increased risk of DF was found under both codominant and dominant genetic models (*P* = 0.002 and *P* < 0.001 respectively).

The findings suggest that genetic predisposition plays a relevant role in the development of DF. Further research is needed to explore the potential use of genetic markers for DF and their implications for public health. This study provides the first insights into the genetic factors associated with DF in the Tunisian population, contributing to our understanding of this prevalent dental condition.

## Introduction

Dental fluorosis (DF) is a developmental defect of tooth enamel and can result in various clinical manifestations, ranging from subtle white marks to more severe pitting and discoloration of the teeth [1, 2]. It’s severity depends on the concentration, duration, and time of exposure to fluoride. The DF is essentially.

Around the world, it was estimated that DF affects particularly Africa, the Eastern Mediterranean and South Asia [3, 4]. In Tunisia, DF is prevalent, particularly in the northern regions [3]. To ensure adequate measurement [4], researchers on DF have shifted to explore it’s underlying genetic origin [5–7]. Single nucleotide polymorphisms (SNPs) [8] is a useful genetic markers to identify genes associated with complex diseases.

The genetic association with DF was widely studied by previous studies [9–11, 5] The systematic review of Gonzalez et al. [5] pointed that a total 15 genes and 18 SNPs were associated were associated to DF. Some SNPs were considered risk factors, while others were considered protective factors [5].

The collagene protein is the most abundant protein in the human body and belongs to family of proteins that strengthen and support many tissues including cartilage, bone, tendon, skin, and the white part of the eye. It has a fundamental role in the bone and soft-tissue formation and presents 78.06% of periodontal ligament fibers, 73.09% of cementum, and 30.50% of alveolar bone [7]. Given the significant role of the collagen type 1 alpha 2 gene (COL1A2) in bone formation, it is plausible that genetic variants in this gene could influence tooth development, particularly in populations exposed to high levels of fluoride. Detecting these genetic variants may serve as a novel biomarker for DF, helping identify individuals at risk This gene induce the pro-alpha2 (I) chain production which is involved in the fabrication of collagen type I, the most abundant form of collagen in the human body [7]. It is about 38 kb and is located at 7q21.3– q22.1 region [12]. Variations in this gene were associated to a numerous pathologies of cartilage, bone and blood vessels. Controversial results were reported about COL1A2 gene and DF [6, 13, 14]. This information could prove invaluable in identifying an individual’s susceptibility and, in turn, modifying their risk of developing DF.

In this terms, no previous study has been performed among the north African population. Thus, the aim of present investigation was to assess the association between the DF and the rs412777 of COL1A2 gene among a Tunisian population.

## Patients and methods

### Study design

It was a case control study established among a Tunisian population from July 2022 to November 2022. Written informed consent was obtained from each patient included in the study.

### Ethical considerations

The research was performed in accordance with ethical guidelines of the 1975 Declaration of Helsinki. The study was reviewed and approved by the ethics committee of the high institute of biotechnology of Monastir, Tunisia (CER-SVS/ISBM 004/2023). Written informed consent was obtained from each patient included in the study.

### Studied population

Two groups were involved The control group included 44 patients without DF They were collected from The blood bank of Fattouma Bourguiba Hospital (Monastir, Tunisia). All of them were blood volunteers’ donors. The case group included 51 patients suffering from DF They were collected from the fixed prosthetic department of the clinic of Monastir, the department of stomatology of the Regional Hospital of Jammel and the blood bank of Fattouma Bourguiba Hospital. For both groups, pregnant women, children, drug addicts and participants aged more than 50 years were excluded. They were well informed about the study protocol and purpose.

### Sample collection

For each subject on both groups, a careful clinical examination was carried on. Then, a detailed clinical file was filled including the gender, the age. The DF was assessed using DEAN [7]. Those with grade 0 and 1 were classified as control group without DF, whereas those who with grade 2 to 5 were classified as case group DNA Extraction and amplification. For each participant of both groups, 3 ml of venous blood was collected in ethylene diamine tetra-acetic (EDTA) to prevent its coagulation (clotting).

#### Polymerase chain reaction (PCR)-based restriction fragment length polymorphism (RFLP)

Genomic DNA was extracted from white blood cells using the salting-out method, as described by Miller et al. in 1988 [15]. Subsequently, genotypes for the variant COL1A2 gene polymorphism were identified using the polymerase chain reaction-restriction fragment length polymorphism (PCR-RFLP) technique. COL1A2 gene polymorphism rs412777 was amplified by PCR (Applied Biosystems (PCRsystem2700), geneAmp ®)using the following primers 5’-GGA AAT ATC GGC CCC GCT GGA AAA-3’(forward) and 5’-GTC CAG CCA ATC CAA TGT TGC C-3’ (Reverse).

The amplification conditions were: 94 °C for 5 min followed by 94 °C/60°C/72°C each one for 30 s for 35 cycles and a final extension at 72 °C for 5 min. Amplicons were digested with PvuII restriction enzyme (Imperial Life Sciences Private Limited, Gurgaon, Haryana, India).

After that, the products were visualized in a 2% agarose gel using a UV transilluminator and gel documentation system. The COL1A2 polymorphism was assessed based on the number and estimated size of the bands compared to the DNA ladder. The presence of only one band of 584 bp indicated the wild genotype (AA). The (AC) or heterozygotes genotype was characterized by the presence of bands at 584 bp, 542 bp, and 42 bp. On the other hand, the (CC) or mutant-homozygotes genotype showed bands at 542 bp and 42 bp. The 42 pb is not observed in the agarose gel because of its low molecular height.

#### Data collection and analysis

To assess the data distribution, the Kolmogorov-Smirnov normality test was employed. Means and standard deviations (SD) were compared using the Student’s t-test. The Pearson’s chi-squared (χ2) test was utilized for the comparison of categorical data in cross tables using SPSS (23). For the comparison of allelic distributions, two-sided independent-sample. Student’s t-tests were performed using Epi Info v.1.4.3 software, developed by the Centers for Disease Control and Prevention in Atlanta, USA.

SNPstats (https://bioinfo.iconcologia.net/SNPstats ) was used to compare the genotype frequencies between the cases and control groups and to obtain odds ratios (OR) with 95% confidence intervals (CI). Dominant, codominant, recessive models were, also, studied. Additionally, to assess if the genotype distributions is conformed to the Hardy-Weinberg equilibrium (HWE), the χ2 goodness-of-fit test was employed, a p-value less than 0.05 suggest a deviation from the HWE. The power calculation for this case-control study was conducted using the Case-Control Study.

Power Calculator (http://sampsize.sourceforge.net/iface/s3.html#ccp). The key parameters included 51 cases, the odds ratio (OR), and the frequency of the minor allele in the control group, all at an alpha risk level of 5%. The resulting overall power of the study was determined to be 77.3%.

## Results

A total of 95 subjects were included (Table 1). Both age and sex were similar among the cases and control group (*p* > 0.05).

**Table 1:** **Demographic characteristics of studied population**

	Case group [*n* = 51]	Control group [*n* = 44]	p
Age	35 ± 11 years.	33 ± 10 years	0.4
Gender			
Males	26	23	0.4
Females	26	21	

We confirmed the adherence to the Hardy-Weinberg equilibrium in our sample with a p-value of 0.064. The allele and genotype frequency distributions of the COL1A2 gene PuvII polymorphism (rs412777) were assessed by analyzing agarose gel results.

The Allelic distribution and frequencies are shown in Table 2. Statistically significant difference was reported between C and A alleles (C vs. A, *P* = 0.001; OR = 0.375 (0.207–0.672) showing that A allele confers protection from DF to carriers.

**Table 2:** Allelic frequencies of rs412777 of COL1A2 gene in the studied population

	All subjects		Control group (44)		Case group (51)		P value	OR (95% CI)
Allele	N	Frequency	N	Frequency	N	Frequency		
**C**	98	0.52	34	0.39	64	0.63	**0.001**	0.37 (0.21–0.67)
**A**	92	0.48	54	0.61	38	0.37		

The distribution of various genotypes exhibited statistically significant differences between the control and case groups (Table 3). Upon conducting Odds Ratio analysis, it was determined that the AA genotype may be considered protective against DF in both codominant and dominant genetic models ((*P* = 0.002; OR = 0.18 (0.06–0.55) and *P* < 0.001; OR = 0.19 (0.07–0.52) respectively)

**Table 3:** Association of COL1A2 gene polymorphism rs412777 with the dental fluorosis under different genetic Models

Genetic Model	Genotype	Control group (44)	Case group (44)	OR (95% CI)	P-value
**Codominant**	C/C	10 (22.7%)	20 (39.2%)	1.00	**0.002**
	A/C	14 (31.8%)	24 (47.1%)	0.86 (0.31–2.34)	
	A/A	20 (45.5%)	7 (13.7%)	**0.18 (0.06–0.55)**	
**Recessive**	C/C	10 (22.7%)	20 (39.2%)	1.00	0.082
	A/C-A/A	34 (77.3%)	31 (60.8%)	0.46 (0.19–1.12)	
**Dominant**	C/C-A/C	24 (54.5%)	44 (86.3%)	1.00	**< 0.001**
	A/A	20 (45.5%)	7 (13.7%)	**0.19 (0.07–0.52)**	

## Discussion

To date, the DF is widespread in Tunisia, especially in South regions. This alteration would affect various hard tissue like the hypomineralization of enamel, dentin hypercementosis, root resorption and hypermineralization of the cementum [16]. For the most severe degrees of DF, it can affect the functionality of the tooth because of the important loss of its structure. It would, also, spoil the esthetic appearance of the individuals, especially for severe situations leading to self-esteem problems. Thus, patients are, increasingly, requiring its esthetic management. The psychological impact could be very important altering the life quality particularly for teenagers and young persons. This height exposure for DF, is certainly, associated to the environmental factors including the intake water. However, several studies have proved a link between genetic and DF. Many studies have established this genetic association between DF and polymorphism [5, 6, 13, 14]. Associated genes, were mainly linked to enamel formation or mineralization [5].

The present investigation pointed the COL 1A2 polymorphism among the Tunisian population. We investigated the COL1A2 polymorphism, specifically rs412777. The differences between genotypes were found to be statistically significant, indicating that AC and CC genotypes were associated with an increased risk of DF. This finding is consistent with a study by Jarquín-Yáñez et al. [17], which included 230 children and reported that the presence of the C allele also increased the risk of DF (OR = 2.59, IC95%: 1.60–4.20, *p* = 0.05). Unlike Escobar-García et al. [18], Saha et al. [19] and Pragya et al [16] Chakraborty et al. [20], did not found associations with the same SNP [18].

Many researchers [7, 16, 17, 21] investigated the SNPs of COL1A2 gene. They confirmed the association between DF and this gene polymorphism. Variable mutations of the concerned gene were explored: Huang et al. were interested to the rs414408 COL 1 A (OR = 4.85, IC95%: 1.22–19.32). As well, Rahila et al. [7] with OR = 31.4; IC95%: 3.9–48.7 and OR = 4.0, IC95%: 1.6–10.1). The divergent results may be attributed to the observed polymorphic sequence which might be influenced by different genetic and environmental backgrounds in different ethnic groups.

According to Rahila et al. [7], this genetic polymorphism was associated to the fluorosis severity. Thus, the comparison within grades of severity of DF was significant (*p* < 0.00125) [7]. However, according to Hung H et al [21], this genetic susceptibility had no influence on the severity of DF This relationship was not explored in this current study.

The sample size, the differences in protocol used or the difference in population characteristics or environmental factors could explain the controversial results.

In the other hand, the COL1A2 mutation was, also, correlated to diverse disorders. In 2003, Willing et al. [22] correlated it to hypomineralisation [7, 22] and decreased bone mineral density. SAHA et al. [19] stated that the effect of COL1A2 polymorphism is related to regulating the bone mineral density. It can be considered, as well, a genetic risk factor related to the of osteoporosis [23].

Taking in consideration all these studies, the COL1A2 polymorphism could be a predictor of many others disorders. The Detection of such genes can be used as a novel biomarker osteoporosis. It can be used in identifying person’s susceptibility and there by alter an individual’s risk of developing DF.

The interaction with environmental factor has been proved and associated to the DF The synergistic risk pattern could explain the genetic–environmental interaction. As there is an individual variability in response to external stimuli which may influence the sensitivity to the DF.

In this terms, some studies have instigated others parameters in their studies. Saha et al. [19] have measured the fluoride concentrations in groundwater, urine, and serum samples. They used a fluoride ion-selective electrode to the fluoride concentrations measurement. High fluoride concentrations water were reported in different regions. The difference was statistically not significant between case and control groups [19]. Pragya et al. [16] have, also, measured the fluoride concentration in the for both groups. Close concentrations were reported with Saha et al. [19]. While Escobar-Garcia D et al. [18] have quantified fluoride concentrations in tap water and urine which were 4.5 ± 0.46 mg/L and 3.1 ± 0.1 mg/L respectively. They compared the reported concentrations between variable genetic genotypes. Non polymorphic individuals (A/A) showed statistically lower water and urine fluoride concentrations severity (*P* = 0.005).

The present study is the first approach of the genetic polymorphism of DF in the Tunisian population. Referring to others surveys conducted in the same topic, the sample size seems to be sufficient [7, 19]. However, comparing to other investigations, it seems to be insufficient [6, 20, 21]. The limitation of the sample size was due to the restricted collection period for participants. Additionally, many people were apprehensive and declined to participate.

In the present, the fluoride exposure of every selected individual was not be accurately ascertained. It would be better if the participants were selected from different regions in order to quantify the fluoride exposure. Measuring the fluoride concentration in the affected regions would help identify the endemic cause of DF.

## Conclusion

The current investigation demonstrated a considerably significant association of COL1A2 ( rs 412,777) polymorphism in the COL1A2 gene with DF among the Tunisian population. The AC and CC genotypes increased the risk of DF. Therefore, genetic predisposition represents a relevant risk factor to develop DF. The detection of such genes can be novel biomarker for DF. Further studies have to be performed in the future.

## Data Availability

The datasets generated and/or analyzed during the current study are not publicly available due to confidentiality reasons but are available from the corresponding author on reasonable request.
